# Hybrid kappa\lambda antibody is a new serological marker to diagnose autoimmune pancreatitis and differentiate it from pancreatic cancer

**DOI:** 10.1038/srep27415

**Published:** 2016-06-08

**Authors:** Mingju Hao, Wenli Li, Lang Yi, Songlin Yu, Gaowei Fan, Tian Lu, Xin Yang, Guojing Wang, Dong Zhang, Jiansheng Ding, Kuo Zhang, Rui Zhang, Guigao Lin, Yanxi Han, Lunan Wang, Jinming Li

**Affiliations:** 1 National Center for Clinical Laboratories, Beijing Hospital, Beijing, People’s Republic of China; 2 Graduate School, Peking Union Medical College, Chinese Academy of Medical Sciences, Beijing, People’s Republic of China; 3 Department of Clinical Laboratory, Qianfo Mountain Hospital of Shandong University, Jinan, People’s Republic of China; 4 China-Japan Friendship Hospital, Beijing, People’s Republic of China; 5 Department of Clinical Laboratory, Peking Union Medical College Hospital, Chinese Academy of Medical Sciences, Beijing, People’s Republic of China

## Abstract

The only generally accepted serological marker currently used for the diagnosis of autoimmune pancreatitis (AIP) is IgG4. Our aim was mainly to determine whether hybrid κ\λ antibody can help to diagnose AIP and to differentiate it from pancreatic cancer. We established an enzyme-linked immunosorbent assay (ELISA) system to measure the levels of hybrid κ\λ antibodies in human sera. Sera were obtained from 338 patients, including 61 with AIP, 74 with pancreatic cancer, 50 with acute pancreatitis, 40 with ordinary chronic pancreatitis, 15 with miscellaneous pancreatic diseases, and 98 with normal pancreas. Our study showed levels of hybrid κ\λ antibodies in the AIP group were significantly higher than in the non-AIP group (*P* < 0.001). The sensitivity, specificity, positive predictive value (PPV) and negative predictive value (NPV) for the diagnosis of AIP were 80.3%, 91%, 66.2% and 95.5% respectively. Furthermore, the combined measurement of serum hybrid κ\λ antibody and IgG4 tended to increase the sensitivity although the difference was not statistically significant (90.2% vs. 78.7%, *P* = 0.08), compared to measurement of IgG4 alone. Our findings suggest that hybrid κ\λ antibody could be a new serological marker to diagnose AIP and differentiate it from pancreatic cancer.

Autoimmune pancreatitis (AIP) is a form of chronic pancreatitis characterized by an autoimmune inflammatory process in which prominent lymphoplasmacytic infiltrates with associated fibrosis of the pancreas cause organ dysfunction^1^. Historically, AIP was used to describe the clinical profiles associated with lymphoplasmacytic sclerosing pancreatitis (LPSP), which is now called type 1 AIP^2^. Accordingly, type 2 AIP is used to describe idiopathic duct-centric chronic pancreatitis (IDCP)^3^. Considering the well-known and long-standing association with serum IgG4 elevation, some experts suggested using “AIP” solely for type 1 AIP and referring to type 2 AIP as IDCP^4^. Steroid therapy often leads to the rapid and sustained resolution of pancreatic mass lesions, pancreatic-duct strictures, and biliary obstruction^1^, and therefore the diagnosis of AIP has a significant impact on the prognosis and treatment of the patient. The characteristic features of dense lymphoplasmacytic infiltration and fibrosis in the pancreas are the gold standard for the diagnosis of AIP. However, it is usually difficult to obtain specimens from the pancreas for histological confirmation^5^.

In 2001, Hamano *et al.*^6^ first reported that serum IgG4 concentrations were highly sensitive and highly specific for AIP. In 2006, IgG4 was first added to the serological criteria for the diagnosis of AIP by the Japan Pancreas Society^7^. The HISORt criteria^8^ and the International Consensus Diagnostic Criteria (ICDC)^2^ allow the use of serum IgG4 as a serological criterion. However, although serum IgG4 is useful for screening, it is not reliable as a single diagnostic marker for AIP. Studies have shown that 4–10% of both healthy controls and controls with other diseases have high serum IgG4 concentrations^9^,^10^. In addition, about 20% of patients with AIP have normal serum IgG4 concentrations at presentation^11^,^12^. The most important disease that should be differentiated from AIP is pancreatic cancer^13^,^14^. Both diseases tend to occur in elderly men, presenting with similar clinical features, such as painless obstructive jaundice, weight loss, and recent-onset diabetes^15^,^16^,^17^. However, many studies reported that moderate elevations in serum IgG4 cannot be used alone to distinguish AIP from pancreatic cancer due to its low sensitivity and specificity^9^,^18^. CA19-9 is considered as a biomarker in pancreatic cancer, but this can also be elevated in other pancreatic diseases such as chronic pancreatitis, or in other pathological states^19^. So far, a simple serological test for the diagnosis and differentiation of AIP from pancreatic cancer is still lacking.

The classic antibody paradigm is that a single mature plasma cell produces symmetrical antibodies composed of one type of immunoglobulin heavy chain and one type of immunoglobulin light chain, either kappa (κ) or lambda (λ)^20^. Within the last 10 years, it has been shown that human IgG4 is a dynamic antibody, which is involved in a continuous process of half-molecule exchange. This process, also referred to as “Fab-arm exchange”, can result in asymmetric antibodies with two different antigen-combining sites^21^,^22^,^23^,^24^. Recently, one report showed that hybrid κ/λ antibodies compose a substantial portion of IgG4 in normal human serum^25^. These molecules are formed by two IgG4 heavy chains plus one κ and one λ light chain. Because AIP is characterized by an elevated serum IgG4 concentration, we attempted to investigate the diagnostic utility of hybrid κ/λ antibody in the diagnosis of AIP and its differentiation from pancreatic cancer.

The principal aim of this study was to establish a light-chain capture sandwich ELISA system to quantify the relative hybrid κ\λ antibody concentrations in patients with a variety of pancreatic diseases. We aimed to determine the sensitivity, specificity and predictive values of serum hybrid κ\λ antibody for the diagnosis of AIP, and compare them with those of serum IgG4. Because the principal differential diagnosis of AIP is pancreatic cancer, we also aimed to evaluate the use of serum hybrid κ\λ antibody and combined measurement with serum IgG4 in differentiating between the two diseases.

## Results

### High prevalence of serum hybrid κ\λ antibodies in patients with AIP in comparison with other study groups

**Figure 1:**
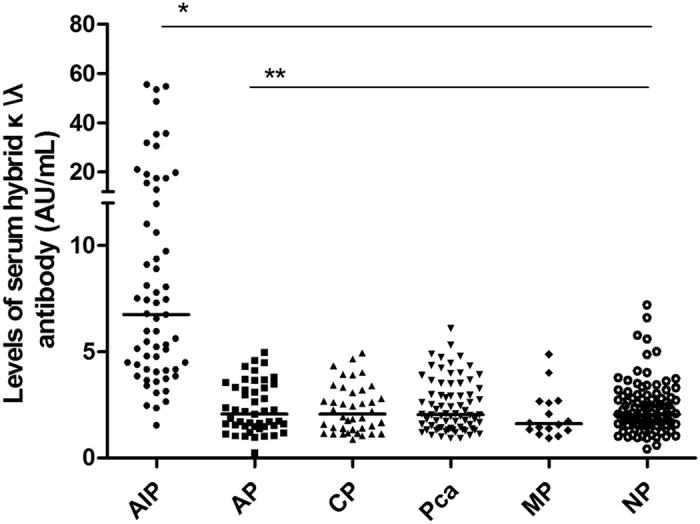
Arbitrary units (AU) of hybrid κ\λ antibody in study groups. Arbitrary units (AU) of hybrid κ\λ antibody in patients with autoimmune pancreatitis (AIP, n = 61), acute pancreatitis (AP, n = 50), ordinary chronic pancreatitis (CP, n = 40), pancreatic cancer (Pca, n = 74), miscellaneous pancreatic diseases (MP, n = 15) and normal pancreas (NP, n = 98). Each horizontal bar represents the median value of serum hybrid κ\λ antibody in each study group. Significance: *P < 0.001 for AIP with respect to each non-AIP group, **P = 0.66 between groups of non-AIP.

After the four-parameter equation of the standard serum was determined, the arbitrary units in test sera were calculated from their O.D. values. Using the double sandwich ELISA system and the standard reference curve we determined the prevalence of hybrid κ\λ antibody in all groups (Fig. 1). The median of hybrid κ\λ antibody in patients with AIP, acute pancreatitis, ordinary chronic pancreatitis, pancreatic cancer, miscellaneous pancreatic diseases and normal pancreas were 6.75 AU/mL (range, 1.53–55.5 AU/mL), 2.06 AU/mL (range, 0.26–4.97 AU/mL), 2.06 AU/mL (range, 0.86–4.94 AU/mL), 2.04 AU/mL (range, 0.93–6.1 AU/mL), 1.61 AU/mL (range, 0.94–4.87 AU/mL) and 2.05 AU/mL (range, 0.42–7.2 AU/mL), respectively. Levels of hybrid κ\λ antibody in AIP group were significantly higher than in the non-AIP groups (*P* < 0.001). There was no significant difference between the non-AIP groups (*P* = 0.66).

### Sensitivity, specificity, and predictive values of hybrid κ\λ antibody in the diagnosis of AIP

**Figure 2:**
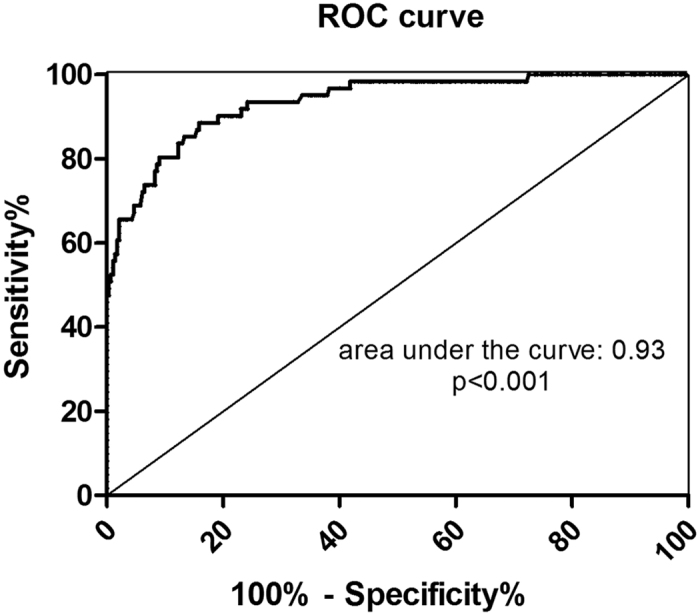
ROC curve evaluating the diagnostic utility for AIP.

In order to evaluate the utility of hybrid κ\λ antibody in the diagnosis of AIP, cutoff values were established to determine the sensitivity and specificity. The sensitivity and specificity of hybrid κ\λ antibody differed at various cutoff values. Using the cutoff value of 4.04 AU/mL from the ROC curve (Fig. 2), we calculated that the sensitivity and specificity were 80.3% (49/61) and 91% (252/277), respectively. The area under the ROC curve was 0.93 (*P* < 0.001). The PPV and NPV of serum hybrid κ\λ antibody for diagnosis of AIP were 66.2% (49/74) and 95.5% (252/264) when calculated using the 61 AIP patients diagnosed during the study period (Table 1).

### Sensitivity, specificity and predictive values of serum IgG4 in the diagnosis of AIP

**Figure 3:**
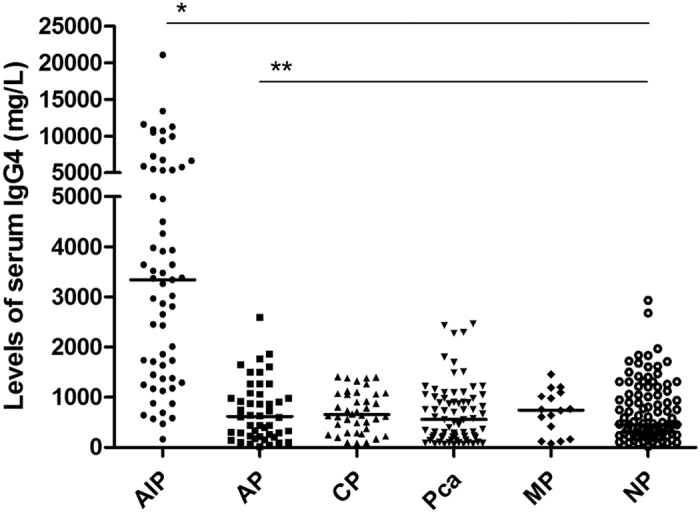
Levels of serum IgG4 in study groups. Levels of serum IgG4 in patients with autoimmune pancreatitis (AIP, n = 61), acute pancreatitis (AP, n = 50), ordinary chronic pancreatitis (CP, n = 40), pancreatic cancer (Pca, n = 74), miscellaneous pancreatic diseases (MP, n = 15) and normal pancreas (NP, n = 98). Each horizontal bar represents the median value of serum IgG4 in each study group. Significance: *P < 0.001 for AIP with respect to each non-AIP group, **P = 0.78 between groups of non-AIP.

The median of serum IgG4 level was 3340 mg/L (range, 164–21100 mg/L), 616 mg/L (range, 22–2594 mg/L), 653 mg/L (range, 81–1409 mg/L), 557.5 mg/L (range, 68–2470 mg/L), 746.5 mg/L (range, 81–1452 mg/L), and 460.5 mg/L (range, 21–2930 mg/L) in patients with AIP, acute pancreatitis, ordinary chronic pancreatitis, pancreatic cancer, miscellaneous pancreatic diseases, and normal pancreas, respectively. Serum IgG4 concentration was significantly higher in patients with AIP than in the other study groups (*P* < 0.001 for each comparison, Fig. 3). When a serum IgG4 concentration of 1350 md/L was used as a cut-off value, the sensitivity was calculated at 78.7% (48/61) with a specificity of 87.7% (243/277). The sensitivity, specificity, PPV and NPV of serum IgG4 were not significantly different to those of serum hybrid κ\λ antibody (78.7% vs. 80.3%, *P* = 0.823; 87.7% vs. 91%, *P* = 0.215; 58.5% vs. 66.2%, *P* = 0.323; 94.9% vs. 95.5%, *P* = 0.777, Table 2).

### The combined measurement of hybrid κ\λ antibody and IgG4 in the diagnosis of AIP

Seropositivity was defined as the elevation of serum IgG4 or hybrid κ\λ antibody levels. For the AIP group, seven patients (7/61, 11.5%) showed elevation of serum hybrid κ\λ antibody with normal serum IgG4, whereas six patients (6/61, 9.8%) showed elevation of serum IgG4 in spite of normal hybrid κ\λ antibody levels (Table 3). As a result, the combined measurement could increase the diagnostic sensitivity from 78.7% to 90.2% (55/61) compared with serum IgG4 alone, although this difference was not statistically significant (*P* = 0.08). The specificity of the combined measurement was not sacrificed significantly (85.9% vs. 87.7%, *P* = 0.53) .

### Serum hybrid κ\λ antibody and IgG4 in the differentiation of AIP from pancreatic cancer

Among patients with pancreatic cancer, nine patients had elevated levels of hybrid κ\λ antibodies (9/74), and eight patients had elevated levels of serum IgG4 (8/74). Hybrid κ\λ antibody (≥4.04 AU/mL) showed a specificity of 87.8%. In the case of IgG4 (≥1350 mg/L), the specificity was 89.2%. There was no significant difference between the two tests (*P* = 0.797, Table 4).

Analogous to the sensitivity in the diagnosis of AIP from all non-AIP groups, the combined measurement showed a specificity of 87.8% (65/74) in the differentiation of AIP from pancreatic cancer, which was almost the same as that (89.2%, 66/74) of IgG4 alone (*P* = 0.797, Table 4).

## Discussion

In a large cohort of patients with a wide variety of pancreatic diseases, we showed that levels of hybrid κ\λ antibodies in patients with AIP were significantly higher than in patients with non-AIP conditions. The sensitivity and specificity for hybrid κ\λ antibody in the diagnosis of AIP were 80.3% and 91% respectively. A proportion (12/61, 19.7%) of AIP patients had normal hybrid κ\λ antibody levels, and a proportion “25/277, 9%” of non-AIP patients had elevated hybrid κ\λ antibody levels. For serum IgG4 levels, the sensitivity and specificity were 78.7% and 87.7%, respectively, which are not as high as the initial study by Hamano *et al.*^26^, but are close to the reports by Ghazale *et al.*^9^ and other previous reports^10^,^11^,^12^. The differences in the diagnostic values of serum IgG4 for AIP could be attributed to differences in the patients enrolled in the studies, or differences in the disease activity of the cases selected^27^,^28^.

Our study showed that the sensitivity and specificity of serum IgG4 was not significantly different from serum hybrid κ\λ antibody in the diagnosis of AIP. In view of this, we wondered whether the combined measurement could increase the diagnostic performance for AIP. Seropositivity was defined as the elevation of serum IgG4 or of hybrid κ\λ antibody. Interestingly, seven AIP patients (7/61, 11.5%) showed elevated levels of serum hybrid κ\λ antibodies despite normal serum IgG4, and six AIP patients (6/61, 9.8%) showed elevation of serum IgG4 with normal levels of hybrid κ\λ antibodies. As a result, the combined measurement of serum IgG4 and hybrid κ\λ antibody could strongly increase the diagnostic sensitivity to 90.2% in comparison with using serum IgG4 alone. More importantly, with the combined measurement the specificity was not significantly sacrificed. This finding is similar to that of a previous report by Song T *et al.*^29^. They showed that the combined measurement of total serum IgG and IgG4 might increase diagnostic sensitivity without compromising the specificity. However, the sensitivity of the combined measurement in their study was only 68.3% (56/82), which was significantly lower than our result (56/82 vs. 55/61, *P* = 0.002).

In our study of 61 patients with AIP, only 12 patients (19.7%) had hybrid κ\λ antibody levels below the cut-off value of 4.04 AU/mL. Therefore, the negative predictive value of hybrid κ\λ antibody levels for AIP was very high (95.5%) and the likelihood of AIP in a patient without hybrid κ\λ antibody elevation is very low, which could help rule out AIP from non-AIP diseases. The positive predictive value of a test is closely related the prevalence of the disease of interest in the studied population. AIP is uncommon with an estimated prevalence of <1/100,000 in the general population^30^. Despite the high specificity of serum hybrid κ\λ antibody for AIP, 91% in our study, it had a low PPV (66.2%). Even so, if more patients with nonspecific abdominal pain were included in our research, the proportion of AIP would be lower and so would the PPV.

When a cutoff value of 4.04 AU/mL of serum hybrid κ\λ antibody was used to differentiate AIP from pancreatic cancer, in contrast to the high prevalence of hybrid κ\λ antibody in patients with AIP, only nine (9/74, 12.1%) patients with pancreatic cancer had elevated levels of hybrid κ\λ antibodies. The specificity was 87.8%, which was almost the same as that for serum IgG4 (89.2%). In keeping with the result for the diagnosis of AIP from all non-AIP groups, the specificity of the combined measurement of serum IgG4 and hybrid κ\λ antibody in the differentiation of AIP from pancreatic cancer was not significantly sacrificed, when compared with that of serum IgG4 alone.

More recently, we reported hybrid κ/λ antibody was a novel biomarker related to disease activity and inflammation in rheumatoid arthritis (RA). Levels of serum hybrid κ/λ antibody were markedly elevated in the patients with RA compared with osteoarthritis (OA) and healthy controls^31^. Nevertheless, the levels of hybrid κ/λ antibody in patients with AIP were significantly higher than those with RA (data not shown). In the present study, all the patients enrolled were suspected to have pancreatic diseases, with obstructive jaundice and abdominal pain, or a pancreatic mass. Hybrid κ/λ antibody is valuable in diagnosing or differentiating AIP from pancreatic cancer based on the clinical findings of suspected pancreatic disorders. Considering the elevation of hybrid κ/λ antibody in RA, or other possible autoimmune diseases, elevated hybrid κ\λ antibody concentrations should be interpreted in conjunction with a thorough evaluation of the clinical, radiological and histological findings for AIP diagnosis, especially for patients who have autoimmune diseases.

Our study has two main limitations. First, the results are limited by a shortage of type 2 AIP patients. Because type 2 AIP is not part of the spectrum of IgG4-related diseases, and does not have definitive serological autoimmune markers^3^, we can speculate that hybrid κ\λ antibody concentrations may be normal in these patients. Nevertheless, this does not change its significance in clinical practice because most cases of AIP in Asia fit the profile of type 1 AIP^2^,^32^,^33^ and patients with type 2 AIP differ significantly in their demography, other organ involvement, and disease relapse^3^. Second, the relatively small number of AIP patients are included that may not be sufficient to allow for a decisive conclusion regarding our results because AIP is an uncommon pancreatic disease. To strengthen the research and pave the way for clinical use of the new biomarker, a prospective study in a different and large patient population is needed in the future.

In summary, this is the first report showing that the level of hybrid κ\λ antibody is markedly elevated in AIP. The high sensitivity and specificity indicated that it could be a new serological marker to diagnose AIP, and to differentiate it from pancreatic cancer. The combined measurement of serum hybrid κ\λ antibody and IgG4 tended to have a higher sensitivity without sacrificing specificity, compared with serum IgG4 alone. Further studies are necessary to clarify its characteristics, and to evaluate the immunoregulatory role in disease activity.

## Methods

### Samples and materials

All 338 patients studied were referred to Peking Union Medical College Hospital for suspected pancreatic disease, with obstructive jaundice and abdominal pain, or a pancreatic mass. Among them, 61 patients (51 men and 10 women) were diagnosed with type 1 AIP and the remaining patients were classified into the five diagnostic groups summarized in Table 5: acute pancreatitis, ordinary chronic pancreatitis, pancreatic cancer, miscellaneous pancreatic diseases, and normal pancreas. Miscellaneous conditions included other pancreatic abnormalities such as pancreatic cyst, pancreatic neuroendocrine tumors, and insulinoma. The diagnosis of AIP was based on the International Consensus Diagnostic Criteria^2^, on the basis of clinical data, imaging, and histopathological findings. All diagnostic cases are definitive cases. Diagnosis of pancreatic cancer was confirmed by pancreatic histology and/or cytology. Patients who had no identifiable pancreatic abnormality were classified as “normal pancreas”. All patients were enrolled randomly between May 2014 and January 2016. Serum samples were stored at −80 C until performing the assay. In addition, a pooled serum sample from 20 healthy subjects (10 men and 10 women) with normal findings on abdominal ultrasonography and without autoimmunity was used as a standard reference serum to determine the standard curve, arbitrarily assigned to contain two units of hybrid κ\λ antibodies per milliliter (AU/mL). The standard reference serum was aliquoted and stored at −80 °C until required. The study was approved by the Ethics Committee of the National Center for Clinical Laboratories and conducted in accordance with the guidelines of the Declaration of Helsinki. Written informed consent for the use of their samples in research was obtained from all subjects.

### Enzyme-linked immunosorbent assay for the detection of serum hybrid κ\λ antibody

The optimum concentrations for each component of the hybrid κ\λ antibody ELISA were determined by a chessboard titration, in which 96-well microplates (Nunc Maxisorp, Denmark) were coated with anti-human λ light-chain antibody (Abcam, China) in 0.05 M sodium carbonate buffer (pH 9.6; 100 μL/ well at 5 μg/mL) and left overnight at 4 °C in a moist box. After a series of washes with PBST (0.5% v/v Tween 20 in PBS), the plates were blocked with 1% w/v BSA in PBST for 2 h at 37 °C.

Next, 100 μl aliquots of test serum at a dilution of 1:10,000 in serum diluent (1% w/v BSA in PBST) were added to all wells. All serum samples were tested in duplicate. Following five washes, the plates were probed with 100 μL/well of mouse anti-κ light-chain HRP-conjugated antibodies (1:2000; Abcam, China) for 1 hour at 37 °C. After extensive washing, the plates were developed with 100 μL/well tetramethyl benzidine (Sigma-Aldrich, USA) for 10 min. The reaction was stopped with 50 μL/well 0.5 M sulfuric acid. Plates were read with a Thermo Multiskan EX plate reader at a wavelength of 450 nm with a 620 nm reference (OD450/620 nm).

The reference standard was used on each ELISA plate to make a standard curve. A twofold serial dilution (1:1000 to 1:128000) of the standard reference serum in dilution buffer was examined in parallel with all samples on each individual plate as described above. The four-parameter logistic-log curve fitting method was used to generate standard curves. Hybrid κ\λ antibody units of the serum samples were then calculated from their O.D. values using the parameters estimated from the standard curve. Linearity and precision (intra- and inter-assay) were carried out to ensure the ELISA performance. Serum samples needed to be re-assayed at a higher dilution when a test sample’s absorbance value fell outside the linear portion of the standard curve.

### Assay of serum IgG4 in all groups

The serum concentrations of IgG4 in all groups were measured using an automated immunonephelometry (BN-II nephelometer, Dade Behring, Germany). Human IgG4 latex reagents (N Latex IgG4, Siemens) were used to quantify the concentrations of serum IgG4. The sensitivity and specificity of serum IgG4 for the diagnosis of AIP were estimated at a concentration of 1350 mg/L, which is the cut-off value used by the Japan Pancreas Society^8^.

### Statistical analysis

ELISA results were analyzed by a four-parameter logistic-log curve fitting program (ELISA v. 2.15; Centers for Disease Control and Prevention, USA) and expressed in arbitrary units (units per milliliter). Sensitivity, specificity, and predictive values were calculated using conventional formulae, and were compared using the χ^2^ test. According to data distribution, values were represented as median and range. Comparisons were performed using the Mann-Whitney U test between AIP and non-AIP group. The Kruskal-Wallis nonparametric analysis is used to compare differences between non-AIP groups. Receiver operator characteristic (ROC) curves were used to judge the diagnostic utility. Statistical analyses were performed with SPSS 19.0 (SPSS Inc., USA) and GraphPad Prism version 6.0 (GraphPad, USA) as appropriate. Two-sided p-values of less than 0.05 were regarded as statistically significant.

## Additional Information

**How to cite this article**: Hao, M. *et al.* Hybrid kappa\lambda antibody is a new serological marker to diagnose autoimmune pancreatitis and differentiate it from pancreatic cancer. *Sci. Rep.* **6**, 27415; doi: 10.1038/srep27415 (2016).

## Figures and Tables

**Table 1:** Sensitivity, specificity and predictive value of hybrid κ\λ antibodies in patients with autoimmune pancreatitis (AIP) and controls (non-AIP).

Levels of serum hybrid κ\λ antibody	AIP (n = 61)	Non-AIP (n = 277)	Total	Predictive value (%)
≥4.04 AU/mL	49	25	74	Positive 66.2%
<4.04 AU/mL	12	252	264	Negative 95.5%
Total	61	277	338	
	Sensitivity 80.3%	Specificity 91%		

**Table 2:** Sensitivity, specificity and predictive value of IgG4 in patients with autoimmune pancreatitis (AIP) and controls (non-AIP).

Levels of serum IgG4	AIP (n = 61)	Non-AIP (n = 277)	Total	Predictive value (%)
≥1350 mg/L	48	34	82	Positive 58.5%
<1350 mg/L	13	243	256	Negative 94.9%
Total	61	277	338	
	Sensitivity 78.7%	Specificity 87.7%		

**Table 3:** The elevation of serum hybrid κ\λ antibody and IgG4 in AIP.

		IgG4 elevation (≥1350 mg/L)	
Hybrid κ\λ antibody elevation (≥4.04 AU/mL)		Yes	No
	Yes	42 (68.8%)	7 (11.5%)
	No	6 (9.8%)	6 (9.8%)

**Table 4:** Sensitivity and specificity of hybrid κ\λ antibody and IgG4 in the differentiation of AIP from pancreatic cancer.

		AIP (n = 61)	Pancreatic cancer (n = 74)	Sensitivity (%)	Specificity (%)
Hybrid κ\λ antibody elevation (≥4.04 AU/mL)	Yes	49	9	80.3%	87.8%
	No	12	65		
IgG4 elevation (≥1350 mg/L)	Yes	48	8	78.7%	89.2%
	No	13	66		
Hybrid κ\λ antibody or IgG4 elevation	Yes	55	9	90.2%	87.8%
	No	6	65		

**Table 5:** Demographic characteristics of study groups.

Study group	n	Age (years)	Sex (male/female)
AIP	61	57 (27–83)	51/10
Pancreatic cancer	74	58 (29–79)	58/16
Acute pancreatitis	50	42 (14–78)	22/28
Ordinary chronic pancreatitis	40	52 (8–78)	28/12
Miscellaneous pancreatic diseases^a^	15	46 (26–77)	6/9
Normal pancreas	98	46 (33–56)	57/41

^a^Miscellaneous pancreatic diseases included 4 patients with pancreatic cysts, 7 patients with pancreatic neuroendocrine tumors, and 4 patients with insulinoma.
